# Computational DNA hole spectroscopy: A new tool to predict mutation hotspots, critical base pairs, and disease ‘driver’ mutations

**DOI:** 10.1038/srep13571

**Published:** 2015-08-27

**Authors:** Martha Y. Suárez, Villagrán, John H. Miller

**Affiliations:** 1 Department of Physics & Texas Center for Superconductivity, University of Houston, Houston, Texas 77204-5005, USA

## Abstract

We report on a new technique, computational DNA hole spectroscopy, which creates spectra of electron hole probabilities *vs*. nucleotide position. A hole is a site of positive charge created when an electron is removed. Peaks in the hole spectrum depict sites where holes tend to localize and potentially trigger a base pair mismatch during replication. Our studies of mitochondrial DNA reveal a correlation between L-strand hole spectrum peaks and spikes in the human mutation spectrum. Importantly, we also find that hole peak positions that do *not* coincide with large variant frequencies often coincide with disease-implicated mutations and/or (for coding DNA) encoded conserved amino acids. This enables combining hole spectra with variant data to identify critical base pairs and potential disease ‘driver’ mutations. Such integration of DNA hole and variance spectra could ultimately prove invaluable for pinpointing critical regions of the vast non-protein-coding genome. An observed asymmetry in correlations, between the spectrum of human mtDNA variations and the L- and H-strand hole spectra, is attributed to asymmetric DNA replication processes that occur for the leading and lagging strands.

Mutations occur in a highly non-random fashion along a DNA molecule. Although natural selection helps shape the DNA’s mutation spectrum—the variant frequency *vs*. nucleotide position—its sequence-dependent physical properties have also been found to locally influence mutation rates^1^,^2^,^3^. Electron holes, in particular, are common targets of base-pair substitutions in cancer and other diseases^1^. A hole is a site of positive charge created when an electron is removed, e.g., by ionizing radiation or contact with an oxidizing compound. The newly created hole then migrates^4^,^5^ until it localizes^6^,^7^,^8^ and potentially triggers a base-pair mismatch during replication^1^.

Hole-induced mutation mechanisms^3^ include tautomeric shift of a hydrogen bond^9^,^10^,^11^ and base deamination^3^,^12^. Guanine has the lowest ionization potential^13^, and thus the highest tendency to trap holes. The hole, alternatively, could affect a neighbouring base, such as the complementary cytosine, or it could localize at another base acting as a potential well if it was closer to the original ionization site. Recent studies^1^,^14^ reveal that mutations in cancer and inherited diseases depend on sequence and disproportionately affect guanine-cytosine (G:C) pairs. DNA’s electrical transport properties^15^ are also affected by sequence^16^, with behaviours ranging from insulating^17^, due to electron or hole localization in a natural sequence, to metallic^18^ or even induced superconducting^19^ in a uniform sequence. Indeed, computed sequence-dependent transport properties of the tumour suppressor gene *p*53 have been found to correlate with point mutation frequencies^20^. Scanning tunnelling microscopy (STM)^21^ confirms the preferential tendency of holes to localize on guanine in natural DNA. Collectively, the above studies are consistent with the idea that DNA’s electronic fingerprint plays a key if not dominant role in driving the underlying mutation probabilities. Nevertheless, natural selection will weed out those mutations that prove highly deleterious or lethal^22^,^23^. The observed spectrum of variants, including single-nucleotide variants (SNVs), will therefore stem from the combined effects of physical mutation mechanisms and natural selection.

The study reported here is intended to: 1) develop a computational method that enables rapid comparisons of hole localization sites on the two DNA strands with peaks in the mutation spectrum; and 2) explore new tools, combining the effects of holes with the pruning effects of natural selection, to identify critical base pairs and likely sites of ‘driver’ mutations, i.e., those which drive a disease state rather than occurring as a ‘passenger,’ e.g., in a growing tumour. Uncovering how DNA’s electronic fingerprint influences its mutation spectrum is of critical importance to genetics and evolutionary biology. A new tool to identity critical base pairs and disease driver mutations could, moreover, pinpoint functionally significant regions of the poorly understood non-coding genome. Recent studies^24^ suggest that 80% or more of non-coding DNA transcribes RNA molecules that play crucial regulatory roles in the organization of complex organisms. This underscores the need to decipher the non-coding genome, biology’s ‘dark matter,’ which makes up more than 97% of the human genome^24^.

Computational DNA hole spectroscopy (D-Spectrum), as proposed here, reveals a DNA molecule’s electronic fingerprint, which can be correlated with the mutational landscape. The spectra for the two strands, of hole probability peaks *vs*. nucleotide position, depict sites where holes tend to localize. These electronic signatures can be correlated with any benign variants, disease mutations, or, for coding DNA, encoded amino acids. DNA hole spectra could, in principle, be obtained experimentally using STM^21^. This could be used, for example, to help validate computational approaches for relatively small DNA molecules. STM, however, is unsuitable for large DNA sequences due to its slowness and the need to optimally place each DNA molecule or segment onto a substrate. The advantage of *computational* DNA hole spectroscopy, by contrast, is that hole spectra can readily be obtained via software from known sequences, eventually encompassing the entire human genome. To the extent that holes enhance the underlying mutation probabilities, the combination of hole and variant data can be used to identify critical base pairs, help differentiate disease driver mutations from passengers, and potentially help identify critical regions of non-coding DNA.

The circular mitochondrial DNA (mtDNA) molecule, with only 16,569 base pairs in humans, has been sequenced for a large number of humans and other organisms, providing a sizeable number of variants that can be used to test and further develop the D-Spectrum algorithm. The two mtDNA strands are the guanine-rich heavy strand (H-strand) and the light strand (L-strand, which is also the reference strand). Mitochondrial DNA is more susceptible to mutations than nuclear DNA, due to mtDNA’s increased exposure to oxidative stress^25^, lack of protective histones, and fewer repair mechanisms^26^. A fraction of the population is thus affected by inherited mitochondrial disorders^26^,^27^, while somatic mtDNA mutations cause or contribute to aging^28^, neurodegenerative disease, cancer, type 2 diabetes, and heart disease^26^. Since it is maternally inherited^29^ and non-recombining, mtDNA provides a potent tool with which to relate DNA’s physical properties to mutations associated with genetic variation, favourable adaptations, e.g., to high altitudes^30^, and disease. The next sections discuss our D-Spectrum algorithm and some tests on mitochondrial DNA.

## Results

### Peaks in the L-strand hole spectrum of mtDNA correlate with peaks in variant frequency

**Figure 1:**
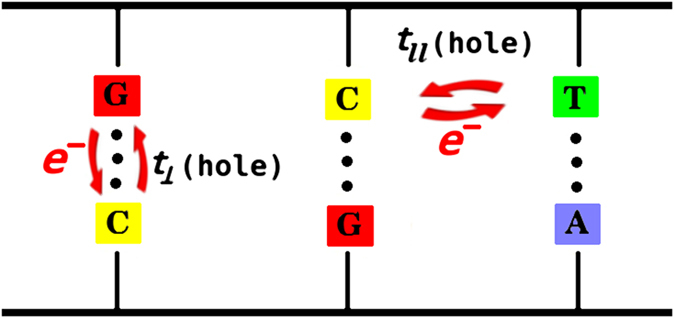
Two-legged ladder model of DNA. A hole can transfer between neighboring bases via the hopping terms *t*_┴_ and *t*_*||*_. On-sites energies are chosen using reported ionization potentials^8^,^13^. (Figure drawn by Martha Y. Suárez Villagrán).

D-Spectrum yields spectra of hole probability peaks on the two strands. In order to study human mtDNA, we model hole localization (see Methods) using the revised Cambridge reference sequence^31^. DNA is represented as a two-legged ladder (Fig. 1)^6^,^7^,^8^, which includes hopping terms that transfer holes between neighbouring bases. The ionization potentials of the bases^8^,^13^ represent hole energy vs. nucleotide position, and the hole probabilities are computed for each energy eigenstate. A uniform DNA molecule, an excellent conductor if doped^19^,^32^, has hole probabilities independent of nucleotide position. The somewhat random energy *vs*. position of naturally occurring DNA, however, leads to one or more sharp peaks depicting hole localization^7^,^8^ for a given energy eigenstate. Putting in a pseudo-thermal distribution of all the states (Methods) then yields complete spectra of hole peaks for the two strands. This study initially focuses on the human mitochondrial gene *ND1*^31^, which encodes the highly conserved ND1 subunit of respiratory complex I^27^,^33^. ND1’s basic function, to convert energy from electrons into mechanical energy to pump protons^27^,^33^, has remained unchanged for more than three billion years. Mutation spectra, as reflected by GenBank (GB) frequencies, and disease mutations are obtained via MITOMAP allele searches^34^.

**Figure 2:**
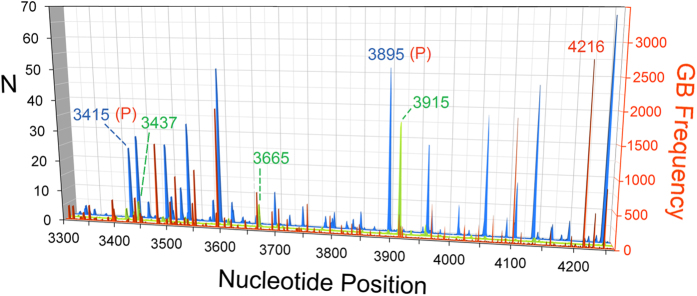
H- and L-strand hole spectra and mtDNA mutation spectrum. **Blue**: H-strand hole probabilities vs. nucleotide position. **Green**: L-strand hole probabilities. *N* = *P/P*_*ave*_ is the number of holes at each site, where *P* is the computed hole probability and *P*_*ave*_ is the average hole probability. **Red-orange**: GenBank (GB) frequencies of human mtDNA variants. H-strand peaks at 3415 and 3895 are both at the 1^st^ codon position for conserved proline’s (P’s)^33^. L-strand hole peaks at positions 3437, 3665, and 3915 are discussed in the text and also labeled in Fig. 3.

**Figure 3:**
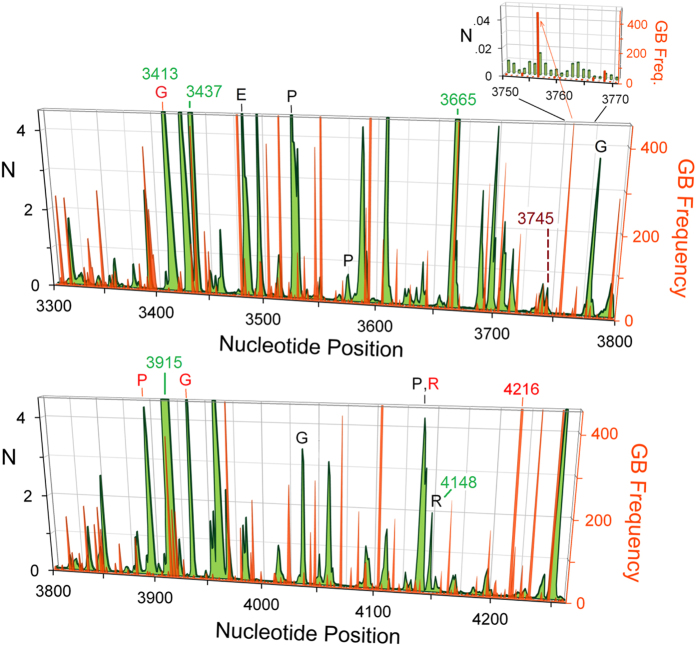
Magnified L-strand hole and human mtDNA mutation spectra. **Green**: Scaled L-strand hole probabilities: *N* = *P/P*_*L*_ is the scaled number of holes at each site, where *P* is the computed hole probability and *P*_*L*_ is the average hole probability for the L-strand alone. **Red-orange**: GenBank (GB) frequency of human mtDNA variations. L-strand peaks at positions 3437, 3665, 3745, and 3915 are example hole peaks correlating with GB frequency spike clusters, while human variations at 3745 and 4216 are high-altitude adaptations^30^. The letters indicate encoded amino acids that are either highly (black) or completely (red) conserved among the species included in a structure-based alignment^33^. (G = glycine, E = glutamic acid, P = proline, R = arginine). The L-strand hole peak at 3413 (encoding G) is shifted slightly from the H-strand peak at 3415.

Figure 2 shows *ND1* hole spectra for the H-strand (blue) and L-strand (green), and the GB frequency (red-orange) *vs*. nucleotide position, depicted for the L-strand by convention^34^. In Fig. 2, the hole spectra are normalized such that *N* would be one hole per base if uniformly distributed among both strands (Methods). The ten largest H-strand hole peaks (blue) dominate the two spectra, and correspond to guanine triplets, quadruplets, or quintuplets. Due the repeated cytosine’s, complementary to guanine, on the reference strand (L-strand), all ten of these peaks correspond to encoded prolines. The two H-strand peaks at 3415 and 3895 correspond to prolines completely conserved among at least 24 species of bacteria and eukaryotes^33^, for which any non-synonymous mutations would likely be deleterious or lethal, and the GB frequency is *zero* at both of these sites. Most of the largest GB frequency peaks are synonymous mutations—benign but providing no advantage. A notable exception is the mutation T4216C, which encodes Y304H (tyrosine → histidine) and has been found to be a possible high-altitude adaptation among Sherpas^30^. Several of the L-strand hole peaks, on cytosine’s, correlate with the large H-strand peaks due to holes hopping from H-strand guanine’s. However, the L-strand peaks are often shifted and/or enhanced by one or more guanine’s directly on the L-strand. The three largest L-strand peaks, at 3437, 3665, and 3915, result from L-strand guanine triplets and a quadruplet (3915 peak). They all correlate with spikes or clusters in variant frequency, as can be better seen in the magnified L-strand plot of Fig. 3. The large L-strand (green) hole peak at 3915, for example, is one of several that engulf entire clusters of mutation spectrum peaks.

The extent to which L-strand hole positions correlate with mutations is further highlighted by zooming in on the L-strand spectrum, normalizing its hole probabilities *P* to the average, *P*_L_, for the L-strand alone, *N* = *P*/*P*_L_, as shown in Fig. 3. Here we see that the overwhelming majority of mutation spectrum peaks (orange-red) occur within, at the edge of, or near a hole peak (green), and that mutation clusters tend to correlate with hole peak clusters. Conversely, gaps in the hole spectrum usually correlate with gaps in the mutation spectrum. Even small hole peaks may influence mutation rates, as suggested by the inset, where mutation peaks at nucleotide positions 3756 and 3768 coincide precisely with small hole peaks at the same positions.

Reproduction and natural selection, over many generations, amplify certain variant populations while suppressing others^22^, as discussed in the next section, which impairs the correlation between hole and mutation peak magnitudes. Hole migration^4^,^5^,^35^, moreover, may account for why GB frequency peaks often occur near the edges, rather than in the middles, of hole peaks. Thus, despite the visually apparent correlations between hole and GB peak positions in Fig. 3, the directly computed Pearson’s correlation coefficient^36^ (*r*-value, see Methods) is quite small, only 0.043, albeit positive. This is substantially improved, however, by limiting GB maxima to 350 to mitigate the amplifying effects of reproduction and natural selection^22^, and by using exponential moving averages of the spectra to account for approximate peak correlations due to hole migration. Using the first procedure alone (capping GB to 350) improves the Pearson’s coefficient to 0.116. Taking exponential moving averages then modifies the L-strand hole and mutation spectra to increase their direct overlap, as shown in Suppl. Fig. 1, which yields a further improved *r*-value of 0.217 over the full nucleotide position range, and 0.297 over the range 3400–4000. By contrast, even with these provisos, the Pearson’s coefficient between modified H-strand hole and mutation spectra is slightly negative, −0.054 over the full range, and −0.036 over the range 3400–4000. (Direct Pearson’s coefficient for H-strand is −0.034.) This striking asymmetry may be due to differences in detailed replication mechanisms for the leading and lagging strands^37^,^38^,^39^,^40^, as will be discussed later.

### L-strand hole peaks not coincident with large variant frequencies often correlate with conserved amino acids and/or disease-associated mutations

Strong L-strand hole peaks that fail to coincide with mutation peaks often occur at codon positions for conserved amino acids, where mutations are likely deleterious or lethal. Figure 3 labels several encoded amino acids that are either highly (black) or completely (red) conserved among the species, ranging from bacteria to humans, included in a structure-based alignment^33^. These coincide with codons at the positions of hole peaks for which the GB frequency of variants is small, consistent with the hypothesis that natural selection has largely prevented such amino acid substitutions from propagating into the gene pool.

**Figure 4:**
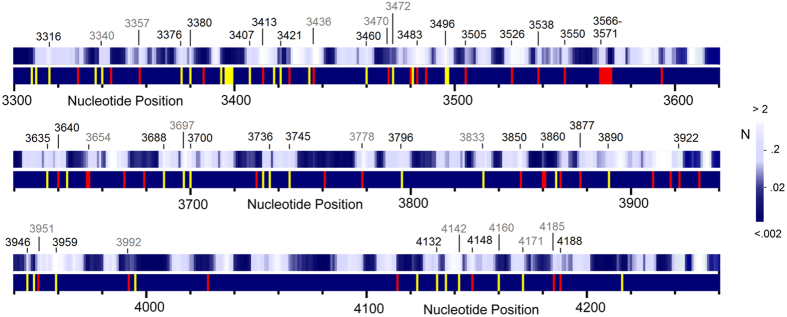
L-strand hole spectrum and disease-implicated mutation sites. **Light bands** (upper strips): L-strand hole peaks. **Yellow bands** (lower strips): Sites of inherited mutations causing or associated with disease. **Red bands** (lower strips): Sites of somatic disease (mostly cancer) associated mutations. Reported disease mutations are obtained from Ref. 14 (mostly somatic) and 33 (mostly inherited) and from MITOMAP^34^ (both inherited and somatic). Numbers above the strips indicate nucleotide positions where disease mutations either match precisely with (**black**), or within one base pair of (**grey**) hole probability maxima, some of which are included in Table 1 and Suppl. Tables 1 and 2.

Table 1 shows a subset of L-strand hole probability maxima that coincide *precisely* with the nucleotide positions of GB frequency peaks and other mutations, including those implicated in various diseases. (Fig. 4 and Suppl. Tables 1 and 2 show more complete sets.) The results suggest that benign (e.g., synonymous), favourable, and deleterious mutations are all potentially influenced by L-strand holes. The mutation G3745A, for example, replacing alanine-147 with threonine, coincides with a hole probability maximum at position 3745. This mutation has been revealed as a possible variant favourable to living in a hypoxic environment at high altitudes^30^. Several mutations in Table 1 and Suppl. Table 1, which coincide with hole peaks but show little or no GB variation frequency, likely due to negative natural selection, replace conserved amino acids and/or are associated with mitochondrial disorders (e.g., MELAS), cancer, and other diseases. For example, G3413A and G4148A, linked to colon adenocarcinoma^14^, replace a highly conserved glycine with aspartic acid, and arginine with histidine, respectively.

Figure 4 shows the positions of inherited (yellow) and somatic (red) disease-implicated mutations (lower bands) and the L-strand hole spectrum (upper bands). Bright regions correspond to high hole probabilities, similar to scanning tunnelling microscope images of DNA^21^. Numbers above the strips indicate precise (black) and approximate (within one base pair, grey) matches between disease mutations and hole probability maxima. Several disease mutations in Fig. 4 and the tables are G-to-A transitions that coincide with or lie near hole maxima but show small GB frequencies due to the deleterious nature of these mutations. However, a few mutations matching or close to hole peaks, e.g., G3316A, T3394C, and C3497T (3316 showing a precise match), are implicated in diseases *and* have high GB frequency counts. These correspond to adult onset diseases or those with sufficiently long-term survival, such as type 2 diabetes, to allow the mutation to be passed on to subsequent generations. This result suggests that mechanisms affecting mutation probabilities, such as holes, skew the effects of natural selection and thus shape evolution. Even harmful mutations could, on occasion, be favoured by the hole mechanism as long as they’re not sufficiently deleterious to be eliminated from the gene pool.

### The H-strand hole spectrum shows no clear correlation with the mutation spectrum

Supplementary Fig. 2 compares the GB frequency peaks with the H-strand hole probabilities, plotted as *N* = *P*/*P*_*H*_, where *P*_*H*_ is the average probability for the H-strand. Here the matches between holes and GB variations appear less visually compelling than those for the L-strand in Fig. 3. This impression is supported by examining Supplementary Tables 2 and 3, which include L- and H-strand hole peaks showing precise matches, correlations within a single base pair, and correlations within several base pairs. Selecting peaks of height *N* ≥ 1 for counting purposes, all 42 (100%) of the L-strand hole peaks for which *N* (= *P*/*P*_*L*_) > 1 are within four base pairs of a GB frequency spike, disease mutation, or cluster of mutations; 39 (93%) are within a single nucleotide site; and 19 (45%) show precise matches. By contrast, of the 34 largest H-strand hole peaks (*N* = *P/P*_*H*_ > 1), none show an exact match to a disease mutation, only 6 (18%) show an exact match to a local GB frequency spike and 8 (24%) are within a single nucleotide position. The relative lack of correlation between H-strand hole and mutation spectra is further corroborated by computing the Pearson’s correlation coefficients for unmodified and modified H-strand hole and GB frequency spectra, following the procedures discussed previously and in Methods. The obtained *r*-values are: −0.034 (full range, unmodified spectra), −0.050 (full range, GB clipped to 350), −0.054 (full range, incorporating both GB capping and moving averages), and −0.036 (same as previous, but over the nucleotide range 3400–4000). In every case the Pearson’s coefficient for the H-strand is small and negative. The Discussion will suggest a hypothesis, based on DNA replication asymmetry between leading and lagging strands, for the different correlations observed when comparing L- and H-strand holes with mutations.

### Constraining L-strand hole peaks with variant data highlights those most likely to correlate with critical base pairs and/or deleterious mutations

We have tested scaling of the hole spectra according to *N’* = *N*/(*Ag* + 1), where *N* is the original hole amplitude, *g* is the GB frequency, and *A* is a scale factor, larger *A* causing greater suppression of amplitudes when they coincide with high variant frequency. This tends to reduce the hole peak widths and has an especially pronounced effect on regions with high variability. Surviving hole peaks for large *A*, where *N’* is not suppressed by the scale factor, are the ones that coincide with small or even *zero* GB variant frequencies. Although such sites lack *germline* mutations, they often coincide with disease (e.g. cancer) implicated *somatic* mutations that occur during aging, for example as seen in MITOMAP^34^, Table S2 of Larman *et al.*^14^, and in Fig. 4. These sites thus likely coincide with deleterious or sometimes lethal mutations, since negative, or purifying selection will tend to eliminate such mutations from the gene pool^22^,^23^.

**Figure 5:**
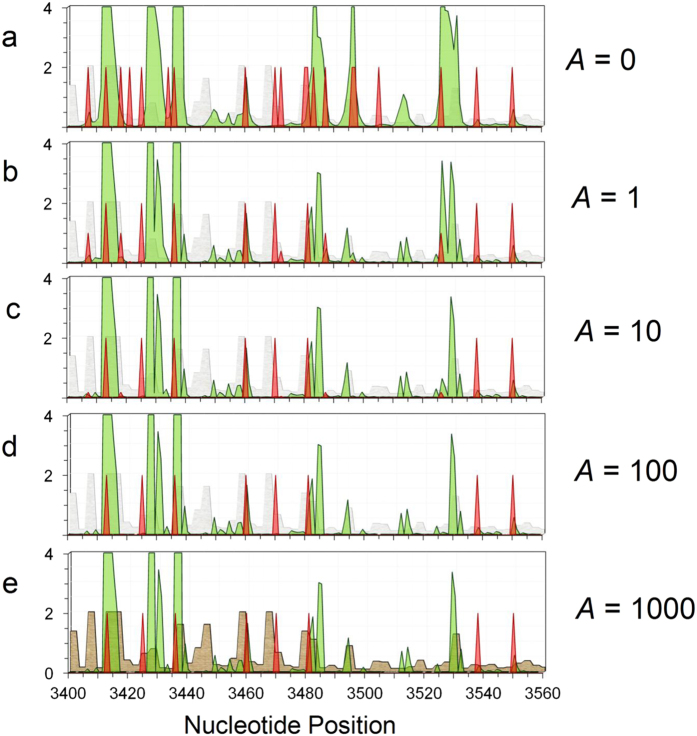
Variant constrained hole (L-strand) spectrum and disease-implicated mutations in the *ND1* segment 3400–3560. **Green**: Variant constrained hole spectrum, *N*′ = *N*/(*Ag* + 1), where *N* = *P*/*P*_*L*_ (see text) and *g* = GB frequency, for various scale factors *A*. **Red-orange**: Variant-constrained disease-associated mutations *m*′ = *m*/(*Ag* + 1) for various scale factors *A*, where *m* = 2 when a disease mutation is present and *zero* otherwise. **Hatched light gray (a**–**d) and brown (e):** Degree of amino-acid conservation (see Methods), where 2 represents complete AA conservation among the 26 species included in an amino acid sequence alignment.

Figure 5 shows such a variant-constrained L-strand hole spectrum for the segment 3400–3560 in *ND1*, for several scale factors *A* in the range 0–1000. It also displays, in red, several variant-constrained disease mutations, *m*’ = *m*/(*Ag* + 1), using the same scale factors *A*, where *m* = 2 (for ease of viewing) when a disease mutation from MITOMAP is present and *zero* otherwise. Those surviving for large *A* are likely to be driver (rather than passenger) mutations since they don’t coincide with normal germline variants. Finally, the hatched light gray and brown plots show the degree of amino acid (AA) conservation, which we define as *C* = 2 exp[-*S*] in Fig. 5 and the factor of 2 is included for ease of viewing. Here *S* represents the information entropy (see Methods)^41^,^42^, which increases with increasing AA variability among the 26 bacteria and eukaryotes, including humans, incorporated in an AA sequence alignment^33^. *C* = 2 represents complete AA conservation when *S* = 0. Suppl. Figure 3 shows similar sets of plots for the *ND1* segment 3860–4000.

Among the eight surviving driver mutations for large *A* in Fig. 5, four coincide with, or are within a base pair of, surviving hole peaks with *N*′∼1.5 or larger. Three additional driver mutations coincide with smaller variant-constrained hole peaks still visible on the graph. Upon examination of the data, we find that the remaining driver mutation at 3470 actually coincides with a local hole maximum too small to be seen in Fig. 5. Most of the remaining variant constrained hole peaks (for large *A*) not coincident with disease mutations, nevertheless, correlate with enhanced AA conservation, where mutations are still likely to be lethal or deleterious. Finally, it has been found that mtDNA transcribes numerous non-coding RNA molecules^43^,^44^,^45^,^46^,^47^, recently dubbed mitochondrial genome-encoded small RNAs, or mitosRNAs^45^. Intriguingly, the variant-constrained hole peak at 3485–3486 lies right at the terminus of a mitosRNA first reported in 2013 (see Table S4 of Reference 45). This suggests that variant constrained hole spectroscopy might be able to identify base pairs critical to both coding and non-coding DNA function.

The mtDNA control region, which contains the D-loop, is the largest non-coding segment of mtDNA, spanning about 1.1 kilobases between the phenylalanine and proline transfer RNA (tRNA) genes. A hypothesis, based on the strand-displacement model of mtDNA replication, is that the D-loop is actually an intermediate of prematurely-terminated H-strand replication. This hypothesis is motivated by the observation that the major 5′ end of the D-loop coincides with the origin of H-strand replication^48^,^49^. Although long known to contain origins of both replication and transcription^50^, other functions of the control region have been a mystery. More recent findings^43^,^45^,^47^ suggest that one of its roles is to transcribe numerous regulatory non-coding RNAs.

Supplementary Figure 4 compares the L-strand hole spectrum with the mutation spectrum for the mtDNA control region. One can see the extremely high degree of variability in the two hypervariable segments^47^, HVS1 (16,024–16,383) and HVS2 (57–372), which are often used in mitochondrial genealogical DNA testing. The region from 16,549 to 120 contains somewhat less variability, however, with the exception of the variant frequency spike of magnitude 19,501 at position 73. This huge variant spike lies right at the edge of the largest hole peak in the entire spectrum. The segment also contains several somatic mutations implicated in cancer, making it an interesting region for which to explore the use of variant-constrained hole spectroscopy.

**Figure 6:**
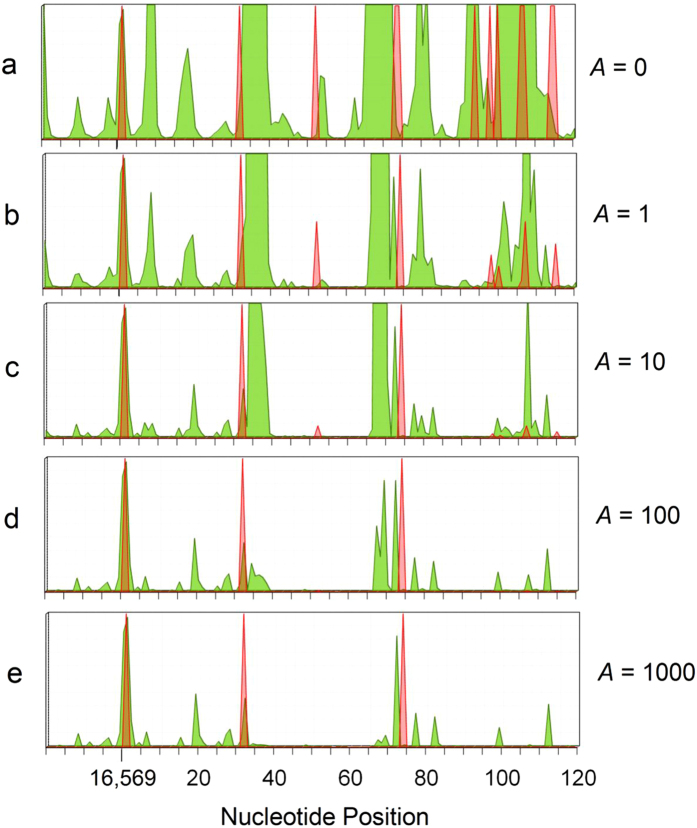
Control region (D-loop) segment 16,549–120, showing variant-constrained hole (L-strand) peaks and disease-implicated mutations. The vertical axes cover the range 0 to 1 in parts a-e. **Green**: Variant constrained hole spectrum, *N*′ = *N*/(*Ag* + 1), where *N* = *P*/*P*_*L*_ (see text) and *g* = GB frequency, for various scale factors *A*. **Red-orange**: Variant-constrained cancer-implicated somatic mutations, *m*′ = *m*/(*Ag* + 1), for various scale factors *A*, where *m* = 1 when a cancer mutation (from MITOMAP) is present and *zero* otherwise.

Figure 6 shows variant-constrained hole peaks and somatic cancer mutations for the segment 16,549–120 of the control region for several values of *A* (ranging from 0 to 1000). The cancer mutations (in red-orange, from MITOMAP) are similarly scaled as *m*′ = *m*/(*Ag* + 1), for various scale factors *A*, where *m* = 1 when a cancer mutation (from MITOMAP) is present and *zero* otherwise. Two of the cancer mutations most likely to be drivers, the only ones surviving when *A* is large, coincide with two of the larger hole peaks in Fig. 6, while the other coincides with a small scaled hole peak adjacent to a larger one. The surviving variant-constrained hole peak (*A* ≥ 100) at position 112 lies near the beginning of the H-strand origin (positions 110–441, Table S6 of Ref. 47), suggesting that a mutation at this location might be deleterious or lethal.

**Figure 7:**
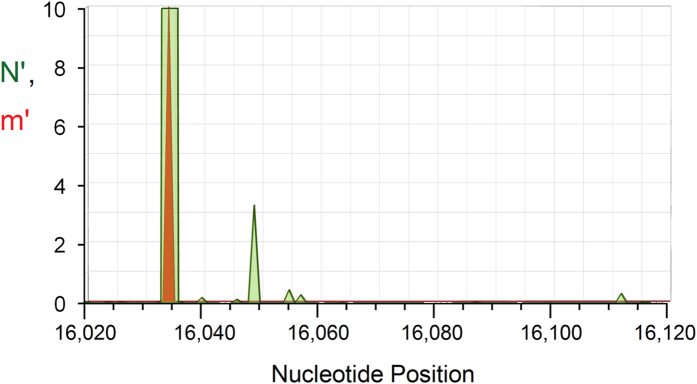
Portion of mtDNA control region, 16,020–16,120, showing variant-constrained hole (L-strand) peaks and ovarian tumor mutation. **Green**: Variant constrained hole spectrum, *N*′ = *N*/(*Ag* + 1), and *g* = GB frequency, for *A* = 1000. **Red-orange**: Variant-constrained cancer-implicated somatic mutations, similarly scaled as *m*′ = *m*/(*Ag* + 1), where *m* = 10 when a cancer mutation (from MITOMAP) is present and *zero* otherwise. Several likely passenger mutations from the allele search are suppressed by the large variant frequencies, leaving only one surviving driver mutation at 16,034.

The method of variant constraint suppresses most disease-associated mutations and hole peaks in the hypervariable segment HVS1, as seen in Fig. 7, which shows the portion 16,020–16,120 using the scale factor A = 1000 for both hole peaks and disease mutations (setting the mutation maximum value to *m* = 10 for ease of viewing). Note, however, that the ovarian tumour mutation, G→A at position 16,034^51^, quite likely a driver mutation, coincides precisely with the largest surviving hole peak in this region. The second largest variant-constrained hole peak, at position 16,049, coincides with somatic G→A mutations seen in aging myocytes^51^ and with an ancient mtDNA variant, 16,049 G→A, extracted from a medieval burial site in North Wales^52^. The segment shown also encompasses two (and part of one additional) recently discovered non-coding mitosRNAs (Table S4 of Ref. 45). One non-coding mitosRNA incorporates the largest hole peak and the ovarian tumour mutation at position 16,034, suggesting a possible regulatory role that becomes disrupted by that mutation.

## Discussion

The results discussed above suggest that: 1) enhanced mutation rates often correlate with hole localization sites; 2) holes on the mtDNA L-strand correlate with mutations much more strongly than those on the H-strand; 3) sites where L-strand hole peaks do *not* coincide with mutation spectrum peaks potentially identify base pairs of critical importance, whose replacements would be lethal or deleterious; and 4) one can potentially identify critical base pairs and disease driver mutations by using variant data to suppress hole peaks and disease mutations when variant frequencies are large.

We hypothesize that the observed striking L/H-strand bias in hole-mutation correlations is due to the asymmetry in DNA replication for the leading and lagging strands. Such replication asymmetry is pervasive among all living organisms since the two strands, aligned in the 3′-to-5′ and 5′-to-3′ directions, run antiparallel to each other^53^. Once a helicase enzyme splits them apart, synthesis via DNA polymerase of each nascent complementary strand can only run in its 5′-to-3′ direction (3′-to-5′ along the parent strand) and can be continuous for the leading strand. During replication of mammalian nuclear DNA, the complementary nucleotides being joined together along the lagging parental strand form segments, known as Okazaki fragments^53^, which are spliced together as the lagging strand finishes its replication process.

The circular mtDNA molecule is replicated by machinery and mechanisms that differ somewhat from that of nuclear DNA^37^,^38^,^39^,^40^. According to the strand-displacement (SD) model^54^,^55^, replication of the leading strand (parental L-strand, daughter H-strand) begins near the origin of H-strand replication (O_H_) within the D-loop. The nascent H-strand is synthesized by the DNA polymerase POLγ along the parental L-strand, displacing and exposing the parental H-strand until it reaches the origin of L-strand replication (O_L_), about two-thirds of the way around the genome. After initiation by an RNA primer, the new L-strand is then synthesized in the opposite direction by POLγ along the parental H-strand to complete lagging strand replication. Although this model has been challenged by competing theories (see Refs 37, 38, 39 for reviews), studies reported in 2014^40^ lend credence to the SD model, revealing that the exposed single parental H-strand (lagging strand) is actually protected by single-stranded DNA binding (SSB) proteins that are released during synthesis of its complementary nascent L-strand.

Our results indicate that, at least for mtDNA, the hole spectrum for the *leading* strand (parental L-strand) shows positive correlation with human variant frequencies and disease mutations. However, the lagging strand (parental H-strand) shows no clear (in fact slightly negative) correlation except when the hole peaks match those of the L-strand. One plausible interpretation is that binding of the exposed H-strand to SSB proteins^40^ alters the hole ionization potentials, and thus the local hole probabilities. Alternatively (or perhaps in addition), any holes created on the original parent double-strand may be sufficiently short lived that only leading strand replication allows them to survive long enough to alter the hydrogen bonds via tautomerization^10^,^11^ to engender incorrect base pairing. Further studies are needed to address this issue, and to determine whether a similar hole-mutation correlation asymmetry between leading and lagging DNA strands exists for the nuclear genome.

Although the study here computes the hole spectrum for the reference mtDNA sequence^31^, D-Spectrum could ultimately be applied to a patient’s personal genome. This would enable it to predict that person’s propensity for specific somatic mutations likely to cause or contribute to cancer or other diseases. A new tool to identity critical base pairs and disease driver mutations, moreover, would help decode the non-coding genome, which makes up more than 97% of the human genome and remains poorly understood. Though such DNA had been labelled as ‘junk’ over a decade ago, the ENCODE project^24^ has recently assigned biochemical functions to about 80% of the genome. This includes the non-protein-coding regions that transcribe RNA molecules^56^, such as micro-RNAs^57^ and long non-coding RNAs^58^, now thought to play crucial roles in the organization of complex organisms. If one uses the analogy to building a house, the coded proteins represent the building blocks, analogous to lumber, bricks, etc. The actual ‘blueprint’ of a complex organism—that which distinguishes a human from a mouse—is likely contained in the vast non-coding genome. Deciphering the non-coding genome may thus prove crucial to decoding the human blueprint itself.

Traditional tools, such as determining protein structures or looking for conserved amino acids, fail for non-coding DNA, so new tools are needed to identify the functionally significant regions. D-Spectrum’s potential for pinpointing which sites are likely to be critical base pairs, and which variants are likely to be disease drivers or lethal mutations, would accelerate the pace of discovery in revealing the various roles of non-coding DNA. Finally, by helping to reveal which bases within transcribed non-coding RNAs, when altered, are likely to have the greatest effects on healthy or cancerous tissue, D-Spectrum could guide the development of new genome-based cancer therapies.

## Methods

This study quantum mechanically models hole localization in human mtDNA using the revised Cambridge reference sequence, GenBank sequence number NC_012920^31^, initially focusing on the mitochondrial gene *ND1*, which includes mtDNA nucleotide sites 3307–4262 on the L-strand^31^. This gene encodes the highly conserved ND1 (Nqo8 or NuoH for *T. thermophilus* or *E. coli*, respectively) subunit of complex I, the largest complex of the electron transport chain. We represent DNA as a two-legged ladder, which includes matrix elements that couple holes between neighboring bases and four different hole-formation energies of the bases, as shown in Fig. 1. This tight-binding method is advantageous due its relative computational simplicity and ability to handle a large number of base pairs in a realistic DNA sequence. Tight-binding models have been extensively refined and validated, for example through comparisons with more complex approaches such as density functional theory, as discussed in a review by Cuniberti *et al.*^6^. Here, the effective tight-binding Hamiltonian is written as^7^,^8^:
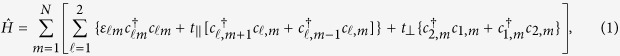
where 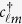 represents a hole creation operator at site *m* on chain 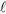. The model includes matrix elements, *t*_*||*_ = 1.0 eV and *t*_┴_ = 0.5 eV^7^,^8^, for hole transfer between nearest neighbors along and between the chains respectively (Fig. 1). The energies 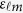 represent on-site hole energies for guanine, cytosine, adenine, and thymine. These are selected using ionization potentials of the respective bases^8^,^13^: *ε*_G_ = 7.75 eV, *ε*_C_ = 8.87 eV, *ε*_A_ = 8.24 eV, and *ε*_T_ = 9.14 eV. For a double-chain representing a segment of mtDNA with *N* sites per chain, the Hamiltonian is treated as a 2*N* × 2*N* matrix, where the various on-site energies are diagonal matrix elements and the hopping terms *t*_*|*|,┴_ are off-diagonal elements. We employ periodic boundary conditions by adding hopping matrix elements that couple the first and last sites of each chain. The eigenenergies *E*_*i*_ and probability amplitudes 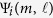 *vs*. site *m* and strand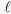 for each eigenstate Ψ_*i*_ are then computed by diagonalizing the Hamiltonian and normalizing the probability amplitudes within the DNA segment of interest.

When using actual sequence data, we find that the lowest ∼20 (out of 1,912 for *ND1*) energy eigenstates Ψ_*i*_ are highly localized, each showing a peak in probability:
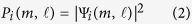
with a single maximum at a nucleotide site *m*, while higher energy eigenstates exhibit a multiplicity of peaks. In order to obtain complete hole spectra for the two strands, we put in a pseudo-thermal distribution of all the states by assuming a Boltzmann distribution:
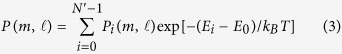
where *N′* = 2*N* is the total number of energy eigenstates, *E*_0_ is the lowest eigenenergy, *k*_*B*_ is Boltzmann’s constant, and *T* is an effective temperature, which may be higher than the actual temperature due to the non-equilibrium nature of hole creation (e.g. by radiation) and transport. We find that an effective, pseudo-thermal energy *k*_*B*_*T* = 0.047 eV incorporates enough energy eigenstates to provide a reasonably complete hole spectrum that compares favorably to mutations. In Fig. 2, hole probabilities are normalized to the average probability *P*_*ave*_ for both strands, *N* = *P*/*P*_*ave*_, such that *N* would be one hole per base for a uniform distribution. In Figs 3, 4, 5, 6, 7 and Supplementary Figures 3–4, *N* = *P*/*P*_*L*_ where *P*_*L*_ is the average hole probability for the L-strand alone, while in Supplementary Fig. 2, *N* = *P*/*P*_*H*_, where *P*_*H*_ is the average probability for the H-strand alone.

The Pearson’s correlation coefficient (*r*-value) is computed using (e.g., see^36^):
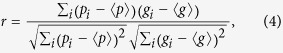
Where *p*_*i*_ and *g*_*i*_ are the hole probability and GB frequency, respectively, at nucleotide site *i* and the mean values are indicated by brackets. Exponential moving averages are computed by summing appropriately normalized values within eight nearest neighbors of the central position *i*, using relative weighting factors: 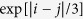.

GenBank frequencies showing human mtDNA variations are obtained by performing allele searches on the MITOMAP website^34^, https://www.mitomap.org/bin/view.pl/Main/SearchAllele, taking the sum if a site has several genetic variations, usually dominated by one mutation. The MITOMAP allele searches also provide inherited and somatic disease-implicated mutations, as do Refs 33 and 14.

The information entropy *S vs*. position for *ND1* is computed, as a measure of variability, from the amino acid (AA) sequence alignment in Suppl. Fig. 1 of Ref. 33, and defined following Ref. 41 (also see^42^ for a review):
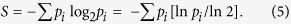
Here *p*_*i*_ represents the fraction of times the *i*^th^ type of AA appears at each position. We find that using *C* =2exp[−*S*] for the degree of conservation highlights the differences between conserved and non-conserved AAs, better than using *C* = 2^*−S*^, in the relevant figures.

## Additional Information

**How to cite this article**: Suárez, M. Y. *et al.* Computational DNA hole spectroscopy: A new tool to predict mutation hotspots, critical base pairs, and disease ‘driver’ mutations. *Sci. Rep.* **5**, 13571; doi: 10.1038/srep13571 (2015).

## Supplementary Material

Supplementary Information

## Figures and Tables

**Table 1:** Selected hole maxima coincident w/GB peaks &/or disease mutations.

L-strand peak	Mutations, Diseases, Adaptations (Subsets shown for mutation clusters)
3316	Cl., G3316A (A4T, GB = 231, NIDDM, LHON, PEO, DM, AML)
3391	Cl., G3391A (G29S, GB = 37); T3394C (Y30H, GB = 367, LHON, CC, DM)
3413	G3413A (G36D, colon adenocarcinoma^14^)
3460	G3460A (A52T, LHON)
3496	Cl., G3496T (A64S, GB = 10, LHON,CC); C3497T (A64V, GB = 93, LHON)
3640	G3640A (A112T, GB = 5), G3640C (A112P, Rectal adenocarcinoma^14^).
3745	Cl., G3745A (A147T, GB = 39, possible adaptive high altitude variant^30^)
3946	Cl., G3946A (E214K, MELAS)
3959	G3959A (G218D, MELAS, colon adenocarcinoma^14^)
4142	G4142A (R279Q, Developmental delay, seizure, hypotonia)
4148	G4148A (R281H, GB = 1, colon adenocarcinoma^14^)

**Left column**: Nucleotide positions of hole probability maxima. **Right column**: Mutations, diseases, & variations are obtained from MITOMAP database^34^ (and cited references) & Ref. 33except where indicated. Cl., cluster of mutations; GB, GenBank frequency; AML, acute megakaryoblastic leukaemia; CC, colorectal carcinoma; DM, diabetes mellitus; LHON, Leber’s hereditary optic neuropathy; MELAS, mitochondrial encephalomyopathy, lactic acidosis, and stroke-like episodes; NIDDM, noninsulin dependent diabetes mellitus; PEO, progressive external ophthalmoplegia.
