# Supplementary material for: Computational DNA hole spectroscopy: A new tool to predict mutation hotspots, critical base pairs, and disease ‘driver’ mutations
Source: Sci Rep. 2015 Aug 27;5:13571. doi: 10.1038/srep13571 (PMC4550865; doi:10.1038/srep13571)
Supplement: Supplementary Information [file srep13571-s1.pdf]

Supplementary Information

Computational DNA hole spectroscopy: A new tool to predict mutation hotspots, critical base pairs, and disease 'driver' mutations

Martha Y. Suárez Villagrán & John H. Miller, Jr.

Supplementary Tables

Supplementary Table 1. Selected L-strand hole maxima coincident with GB peaks, disease mutations, &/or adaptations.

L-strand peak	Mutations, Diseases, Adaptations, Subsets of Mutation Clusters
3316	Cl., G3316A (A4T, GB=231, NIDDM, LHON, PEO, DM, AML)
3391	Cl., G3391A (G29S, GB=37); T3394C (Y30H, GB = 367, LHON, CC, DM)
3413	G3413A (G36D, colon adenocarcinoma, Ref. 14)
3460	G3460A (A52T, LHON)
3483	G3483A (syn: E59E, GB=98); G3481A (E59K, PE, MELAS)
3496	Cl., G3496T (A64S, GB=10, LHON,CC); C3497T (A64V, GB=93, LHON)
3513	Cl., C3513T (syn: T69T, GB=54); C3516A (syn: L70L, GB=805)
3531	Cl., G3531A (syn: P75P, GB=120)
3640	G3640A (A112T, GB=5), G3640C (A112P, Rectal adenocarcinoma, Ref. 14).
3688	Cl., G3688C (A128P, GB=12); G3688A (A128T, LS, ME)
3745	Cl., G3745A (A147T, GB=39, possible adaptive high altitude variant, Ref. 30)
3796	Cl., A3796T (T164S, GB=128); A3796G (T164A, GB = 157, AOD)
3834	Cl., G3834A (L176L, GB=173); T3833A (L176Q, PEG)
3882	Cl., G3882A (syn: E192E, GB=99)
3915	Cl., G3915A (syn: G203G, GB=362); G3918A (syn: E204E, GB=178)
3946	Cl., G3946A (E214K, MELAS)
3959	G3959A (G218D, MELAS, colon adenocarcinoma (Ref. 14))
4059	Cl., C4059T (syn: S251S, GB=31); T4062C (syn: P252P, GB=17)
4092	Cl., G4092A (syn: K262K, GB=66); A4093G (T263A, GB=70)
4132	Cl., G4132A (A276T, GB=6, NAION associated)
4142	G4142A (R279Q, Developmental delay, seizure, hypotonia)
4148	G4148A (R281H, GB=1, colon adenocarcinoma, Ref. 14)
4197	Cl., C4197T (syn: T297T, GB=165)

Left column: Nucleotide positions of 23 hole probability maxima. **Right column**: Mutations, diseases & variations obtained from MITOMAP database[34] (w/ extensive references) & Ref. 33, except where otherwise indicated. Cl., cluster of mutations; GB, GenBank frequency; AML, acute megakaryoblastic leukaemia; AOD, adult onset dystonia; CC, colorectal carcinoma; DM, diabetes mellitus; LHON, Leber's hereditary optic neuropathy; LS, Leigh syndrome; ME, mitochondrial encephalomyopathy; MELAS, mitochondrial encephalomyopathy, lactic acidosis, and stroke-like episodes; NAION, non-arteritic anterior ischemic optic neuropathy; NIDDM, noninsulin dependent diabetes mellitus; PE, progressive encephalomyopathy; PEG, pseudoexfoliation glaucoma; PEO, progressive external ophthalmoplegia.

Supplementary Table 2. Correlation of L-strand hole peaks (N > 1) with mutations.

L-strand peaks	$N = P/P_L$	**Mutations, Diseases, Adaptations** (Subsets shown for mutation clusters.)
**3316**	**1.75**	**Cl., G3316A (A4T, GB = 231, NIDDM, LHON, PEO, DM, AML)**
**3391**	**2.56**	**Cl., G3391A (G29S, GB = 37); G3391C (G29C, GB = 4); T3394C (Y30H, GB = 367, LHON, CC, DM)**
**3413**	**13.85**	**G3413A (G36D, COAD, Ref. 14)**
**3428**	**10.65**	**Cl., C3429T (syn: G41G, GB = 3)**
**3437**	**23.38**	**Cl.,: G3438A (syn: G44G, GB = 419); G3438C (syn: G44G, GB = 1); G3436A (G44R , Head, neck tumor)**
**3460**	**1.64**	**G3460A (A52T, LHON)**
**3483**	**5.78**	**G3483A (syn: E59E, GB = 98, COAD (Ref. 14, passenger mutation)); G3481A (E59K, PE, MELAS)**
**3496**	**5.81**	**Cl.,: G3496T (A64S, GB = 10, LHON, CC); C3497T (A64V, GB = 93, LHON); A3480G (PT)**
**3513**	**1.07**	**Cl.,: C3513T (syn: T69T, GB = 54); C3516A (syn: L70L, GB = 805)**
**3526**	**6.79**	**G3526A (A74T, GB =1); C3525A (syn: T73T, GB = 18)**
**3531**	**3.71**	**Cl., G3531A (syn: P75P, GB = 120)**
**3574**	**4.17**	**C3573A (syn: L89L, GB = 5); C3573T (syn: L89L, GB = 4); C3576T (syn: P90P, GB = 9)**
**3587**	**4.27**	**C3588T (syn: P94P, GB = 9); C3588A (syn: P94P, GB =1)**
**3608**	**7.93**	**A3606G (syn: L100L, GB = 164); C3609T (syn: G101G, GB = 4)**
**3640**	**1.09**	**G3640A (A112T, GB = 5), G3640C (A112P, READ, Ref. 14).**
**3665**	**27.42**	**Cl. (mostly syn), including G3666A (syn: G120G, GB = 609)**
**3688**	**2.88**	**Cl., G3688C (A128P, GB = 12); G3688 (A128T, LS, ME)**
**3698**	**4.37**	**G3697A (G131S, MELAS, LS, LDYT)**
**3707**	**2.16**	**G3705A (syn: L133L, GB = 356); A3708G (syn: R134R, GB = 2)**
**3715**	**1.52**	**G3715C (A137P); A3714C (syn: V136V, GB =2)**
**3779**	**3.17**	**Cl., C3780T (syn: G158G, GB = 29); T3777C (syn: S157S, GB = 44)**
**3834**	**1.18**	**Cl., G3834A (L176L, GB = 173); T3833A (L176Q, PEG)**
**3850**	**2.54**	**Cl., G3850C (A182P, GB = 1); G3849A (syn: L181L, GB = 117); T3847C (syn: L181L, GB = 175)**
**3882**	**1.12**	**Cl., G3882A (syn: E192E, GB = 99)**
**3895**	**4.30**	**C3897T & C3897A (both syn: P197P, GB = 5 & 2, respectively); C3894T (syn: T196T, GB = 10); G3890A (R195Q, PE, LS, OA,)**
**3915**	**131.24**	**Cl.,: G3915A (syn: G203G, GB = 362); G3918A (syn: E204E, GB = 178, breast tumor passenger mutation)**
**3934**	**5.52**	**A3933G (syn: S209S); G3935A (G210D, GB = 1); C3936T (syn: G210G, GB = 8); C3936A (syn: G210G, GB = 1); T3931C (S209P, head/neck tumor)**
**3952**	**1.50**	**Cl. (GB up to 15), including: T3949C (E214K, MELAS, thyroid oncocytoma); C3951T (pituitary carcinoma).**
**3955**	**1.75**	**C3954T (syn: A216A, GB = 15); A3957G (syn: A217A, GB = 4)**
**3959**	**12.58**	**G3959A (G218D, MELAS, ref. 33, MITOMAP; COAD, Ref. 14)**
3967	2.32	Cl., C3966T (syn: F220F, GB=3); C3969T (syn: A221A, GB=25); C3970T (syn: L222L, GB=551)
**3982**	**1.43**	**A3981G (syn: M225M, GB=140)**
**3985**	**1.63**	**Highly conserved glutamic acid. Adjacent: C3990T (syn: Y228Y, GB=78); C3992T (T229M, GB=212).**
4036	3.40	A4038G (syn: G244G, GB = 34); T4028C (somatic, MNGIE fibroblasts)
**4059**	**3.11**	**Cl., C4059T (syn: S251S, GB = 31); C4059A (syn: S251S, GB = 1); T4062C (syn: P252P, GB = 17)**
**4092**	**1.06**	**Cl., G4092A (syn: K262K, GB =66); A4093G (T263A, GB = 70)**
4110	1.51	Cl., T4114C (F270L, AML, Ref. 14); A4104G (syn: L266L, GB = 1909); C4106T (T267I, GB = 1); C4107T (syn: T267T, GB = 18); C4109T (S268F, GB = 3); C4111T (syn: L269L, GB = 2) G4113A (syn: L269L, GB = 49)
**4139**	**4.22**	**Cl., C4140T (syn: P278P, GB = 34); A4136G (Y277C, GB = 33, LHON)**
**4142**	**3.04**	**G4142A (R279Q, Developmental delay, seizure, hypotonia)**
**4148**	**1.97**	**G4148A (R281H, GB = 1, COAD, Ref. 14)**
**4197**	**3.61**	**Cl., C4197T (syn: T297T, GB = 165)**
**4251**	**5.39**	**Cl., C4251T (syn: P315P, GB = 1); T4248C (syn: I314I, GB = 902)**

<u>Underlined & bold</u>: Exact match to disease or spike in GB frequency. **Bold**: Correlates within one nucleotide position. **Left column**: Nucleotide positions of hole probability maxima. **Middle column:** Maximum heights of hole probability peaks. **Right column**: Mutations, diseases, & variations listed in the MITOMAP database[34] (w/ extensive references) & Ref. 33, except where otherwise indicated (e.g., Ref. 14). **Cl**., cluster of mutations; **GB**, GenBank frequency; **AML**, acute megakaryoblastic leukaemia; **AOD**, adult onset dystonia; **CC**, colorectal carcinoma; **COAD**, colon adenocarcinoma; **DM**, diabetes mellitus; **LDYT**, Leber's optic atrophy & dystonia; **LHON**, Leber's hereditary optic neuropathy; **LS**, Leigh syndrome; **ME**, mitochondrial encephalomyopathy; **MELAS**, mitochondrial encephalomyopathy, lactic acidosis, and stroke-like episodes; **MNGIE**, Mitochondrial neurogastrointestinal encephalomyopathy; **NAION**, non-arteritic anterior ischemic optic neuropathy; **NIDDM**, noninsulin dependent diabetes mellitus; **PE,** progressive encephalomyopathy; **PEG**, pseudoexfoliation glaucoma; **PEO**, progressive external ophthalmoplegia; **PT**, prostate tumor; **READ**, Rectal adenocarcinoma.

Supplementary Table 3. Correlation of largest H-strand hole peaks ($N > 1$) with mutations.

H-strand peaks	$N = P/P_H$	**Mutations, Diseases** (Subsets shown for mutation clusters.)
3324	1.863	Cl., C3330T (syn: L8L, GB=51); T3329C, L8P, OV, S4
3341	1.204	Cl., A3339G (syn: V11V, GB=3); C3342T (syn: P12P, GB=14)
3415	13.6703	G3413A (G36D, colon adenocarcinoma[14])
3430	15.9914	Cl., C3429T (syn: G41G, GB=3); C3432T (syn: P42P, GB=1)
3449	3.224	Cl., A3447G (syn: Q47Q, GB=162), C3448G (P48A, GB=0), C3450T (syn: P48P, GB=111)
3485	14.5835	Cl., C3486T (syn: P60P, GB=14), C3487A (L61M, GB=1), T3488C (L61P, GB=1), T3488A (L61Q, GB=1), A3489G (syn: L61L, GB=1)
3494	4.4725	Cl., C3495A (syn: P63P, GB=13); G3496T (A64S, GB=10); G3496A (A64T, GB=7)
**3513**	**6.3478**	**Cl., C3513T (syn: T69T, GB=54)**
3528	18.6788	Cl., G3531A (P75P, GB=120); C3528T (syn: A74A, GB=12); C3527G (A74G, GB=1); G3526A (syn: A74T, GB=1); C3525A (syn: T73T, GB=18); C3525T (syn: T73T, GB=1)
3575	4.149	Cl., C3573T (syn: L89L, GB=4); C3573A (syn: L89L, GB=5); C3576T (syn:P90P, GB=9)
3587	29.1458	Cl., C3588T (syn: P94P, GB=9); C3588A (syn: P94P, GB=1); C3589T (syn:L95L, GB=1); C3589G (L95V, GB=1)
3610	3.985	C3609T (syn: G101G, GB=4)
**3630**	**1.2211**	**C3630T (syn: T108T, GB=55)**
3655	1.7063	Cl., A3652G (I116V, GB=4); T3653A (I116N, GB=1); C3654T (syn: I116I, GB=10)
3690	6.3346	Cl., C3690T (syn: A128A, GB=2); C3690G (syn: A128A, GB=0); C3691T (syn: L129L, GB=6); G3688C (A128P, GB=12)
3717	2.0609	Cl., incl. A3720G (syn: Q138Q, GB=192)
**3741**	**3.843**	**C3741T (syn: T145T, GB=75)**
3782	2.1785	Encoded conserved serine; hole peak straddled by GB frequency peaks.
3798	3.0347	Cl., C3820T (L172F, GB=2); C3822T (syn: L172L, GB=1); C3819T (syn: H171H, GB=50)
3820	1.4489	Cl., C3798T (syn: T164T, GB=5); C3797G (T164S, GB=1); A3796G (T164A, GB=157); A3796T (T164S, GB=128); C3795T (C163T, GB=1)
3832	1.8597	Cl., C3831T (syn:L175L, GB=2); C3831A (syn: L175L, GB=2); C3832A (L176M, GB=3); G3834C (syn: L176L, GB=3); G3834A (syn: L176L, GB=173)
3845	3.3967	Cl., C3846T (syn: P180P, GB=1); T3847C (syn: L181L, GB=175)
3895	29.8182	C3894T (syn: T196T, GB=10)
**3954**	**1.0808**	**Cl., C3954T (syn: A216A, GB=15); C3952T (A216T, GB=1)**
3961	16.2256	C3960T (syn:G218G, GB=7)
**3969**	**4.3261**	**Cl.,: C3969T (syn: A221A, GB=25); C3970T (syn: L222L, GB=551)**
4014	5.5771	Cl., C4014T (syn: T236T, GB=2); C4015T (L237F, GB=2); C4013T (T236I, GB=13); A4012G (T236A, GB=27)
**4059**	**21.9179**	**Cl., C4059A (syn: S4059S, GB=32)**
4095	3.6409	Cl.,: C4095T (syn: T263T, GB=8); C4094T (T263I, GB=3); C4096T (syn: L264L, GB=2); A4093G(T263A, GB=70)
4110	10.0778	Cl., C4109T (S268F, GB=3); C4111T (syn: L269L, GB=2)
4139	27.2915	C4140T (syn: P278P, GB=34)
**4170**	**2.847**	**C4170T (syn: L288L, GB=40); Cl.,: C4170A (syn: L288L, GB=1); C4171T(L289L, GB=5); T4172A (L289Q, GB=21)**
**4197**	**3.607**	**Cl.,: C4197T (syn: T297T, GB=165); C4198T (syn: T298T, GB=41); A4200T (syn: L298L, GB=14); A4200G (syn: L298L, GB=6)**
4251	39.1075	Cl., C4251T (syn: P315P, GB=1); T4248C (syn: I314I, GB=902)

Underlined & bold: Exact match. **Bold**: Correlates within one nucleotide position of local maximum in GB frequency (10 or greater) from the MITOMAP database. **Left column**: Nucleotide positions of hole probability maxima. **Middle column:** Maximum heights of hole probability peaks. **Right column**: Mutations, diseases, & variations listed in the MITOMAP database[34]. **Cl.**, cluster of mutations; **GB**, GenBank frequency.

Supplementary Figures

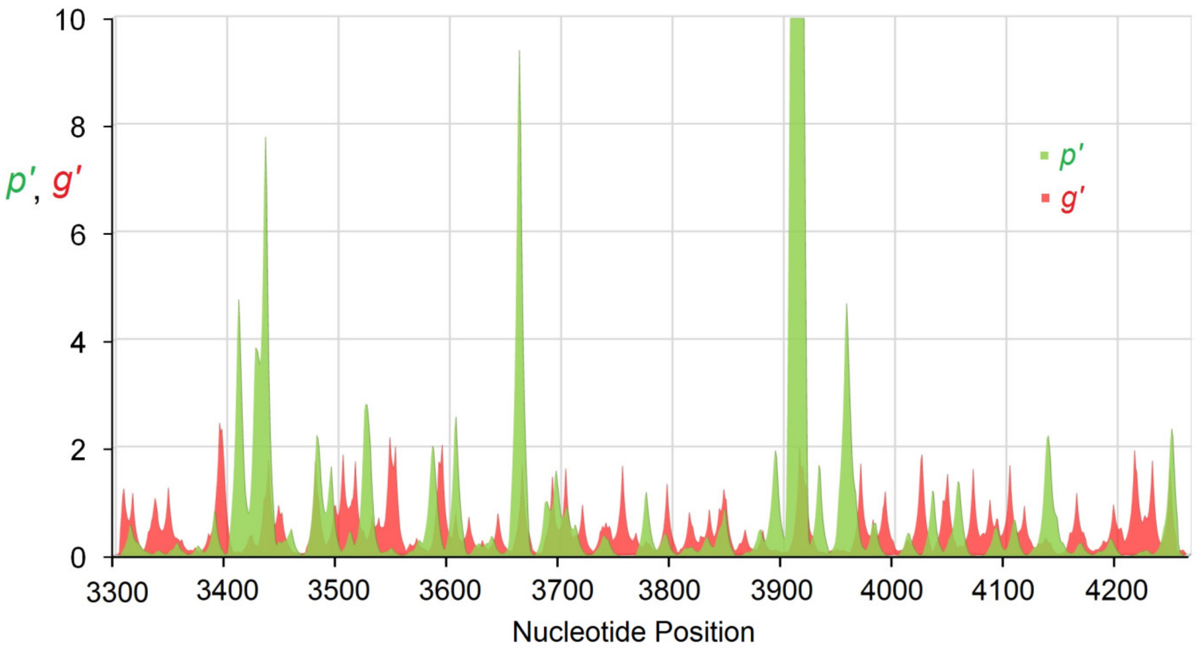

Supplementary Figure 1. Exponential moving averages for L-strand hole spectrum & mutation spectrum for mtDNA gene *ND1*. Green: Exponential moving average, p', of L-strand hole probabilities scaled to their average value, P/P_L. **Orange-red**: Mutation spectrum, showing exponential moving average, g', of scaled mtDNA GenBank frequencies (capped to 350 and scaled as GB freq./40 before taking moving average). The overlap yields a Pearson correlation coefficient of 0.217 over the full range and 0.297 over the range 3400-4000. (See main text and Methods for discussion.)

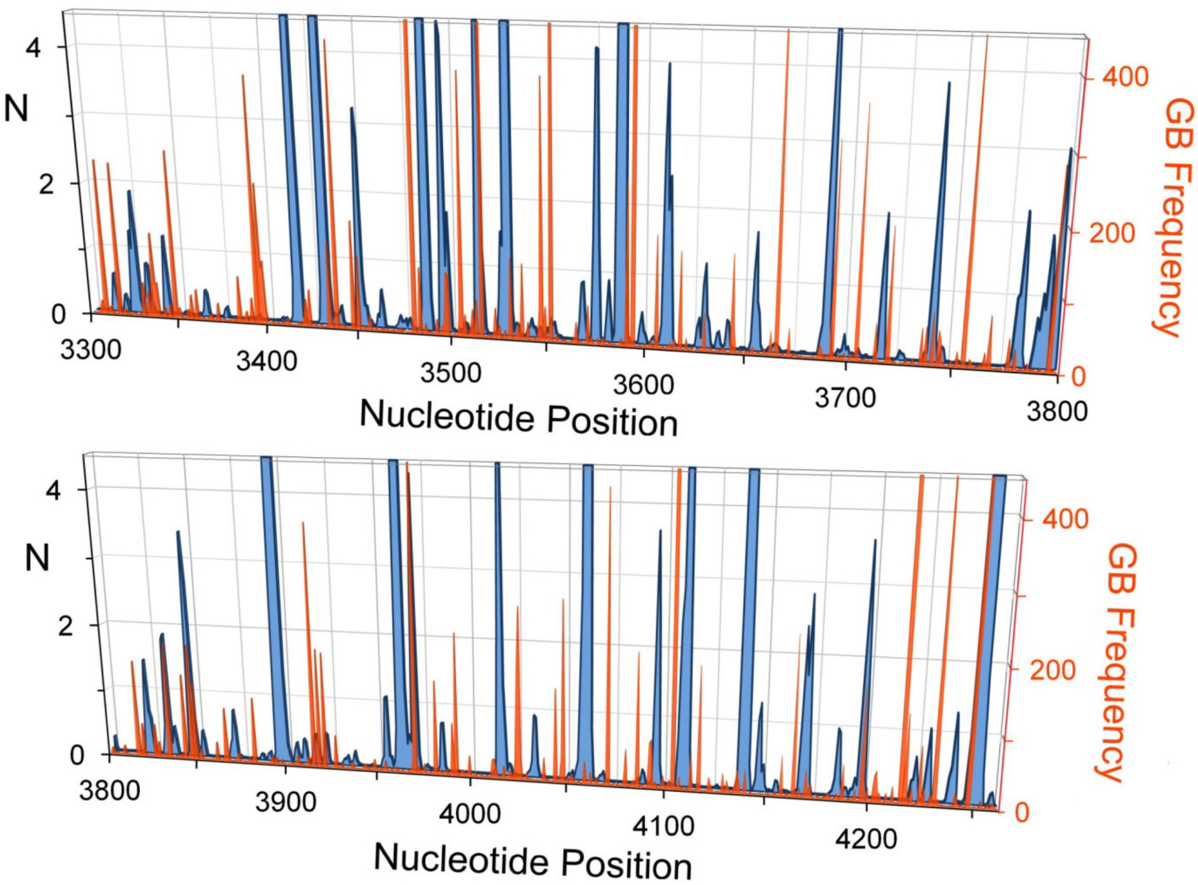

Supplementary Figure 2. H-strand hole spectrum & mutation spectrum for mtDNA gene *ND1*. **Blue**: Scaled H-strand hole probabilities: $N = P/P_H$ is the scaled number of holes at each site, where P is the computed hole probability and P_H is the average hole probability for the H-strand alone. **Orange-red**: Mutation spectrum, showing mtDNA GenBank frequencies. The Pearson correlation is small and negative, even when capping GB freq. to 350 and taking moving averages. (See main text and Methods for discussion.)

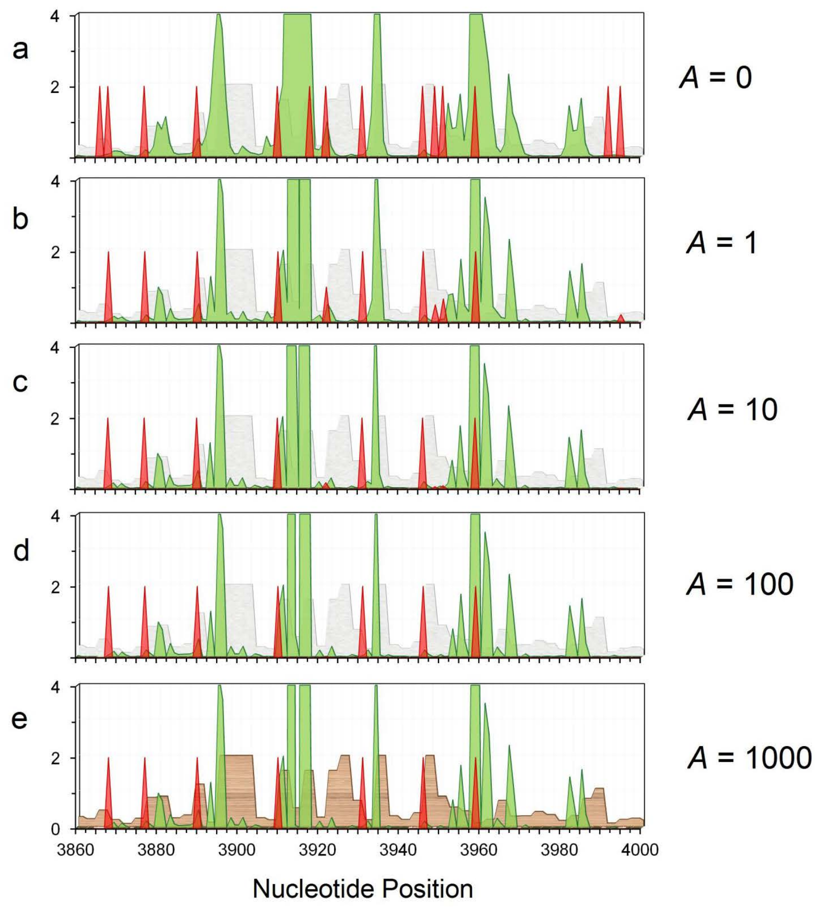

Supplementary Figure 3. Variant constrained hole (L-strand) spectrum and disease-implicated mutations in the *ND1* segment 3860-4000. Green: Variant constrained hole spectrum, $N' = N/(Ag+1)$, where $N = P/P_L$ (see text) and g = GB frequency, for various scale factors A. Surviving hole peaks for large A are the ones most likely to coincide with deleterious or even lethal mutations. **Red**: Variant-constrained disease-implicated mutations $m' = m/(Ag+1)$ for various scale factors A, where $m = 2$ (for ease of viewing) when a disease mutation (from MITOMAP) is present and *zero* otherwise. Those surviving for large A are most likely to be driver mutations. **Hatched light gray (a-d) and brown (e):** Degree of amino-acid conservation (see text), where 2 represents complete AA conservation among the 26 species included in an amino acid sequence alignment.

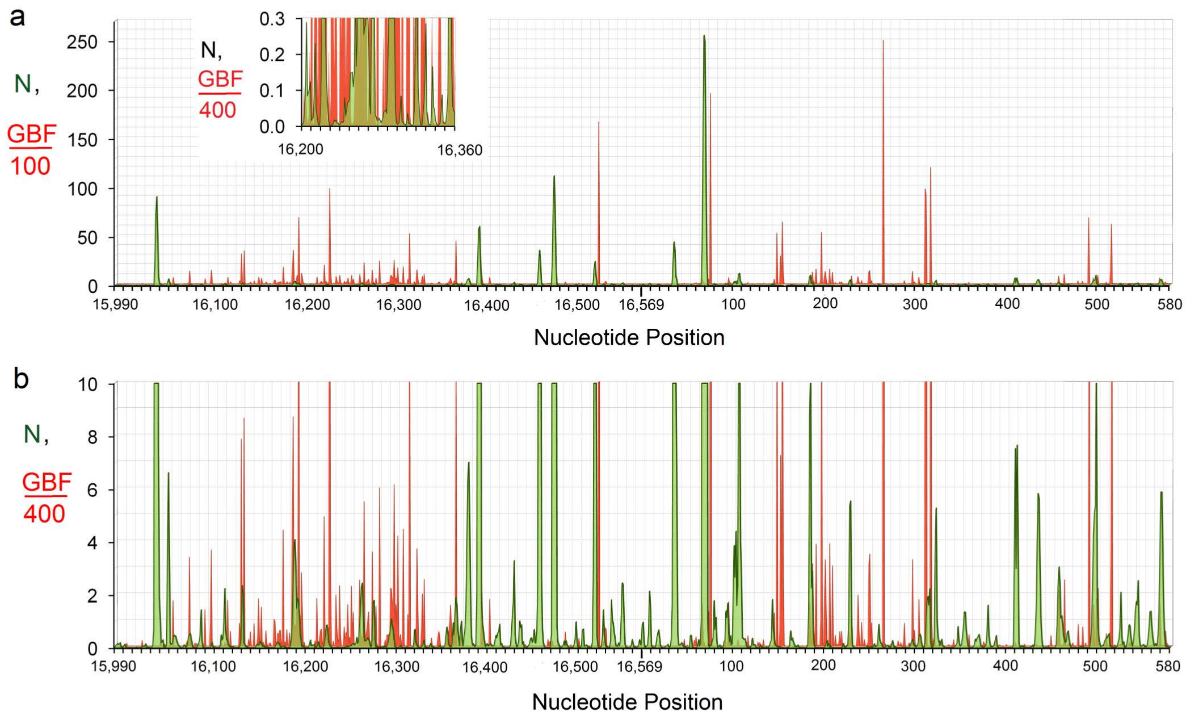

Supplementary Figure 4. Mitochondrial DNA control region (D-Loop) hole spectrum for the L-strand. **Green:** Scaled L-strand hole probabilities: *N* is the scaled number of holes at each site (see text). **Orange-red:** GenBank frequency (GBF) of variants vs. position. **a)** Overview of hole spectrum, where GB frequencies are reduced by a factor of 100**. b)** Magnified view of hole peaks as compared to variant frequencies (GBF/400)**. Inset to a):** Greatly magnified view of hole spectrum for the interval 16200-16360 (showing GBF/400), revealing several small hole peaks that appear to correlate with mutations and clusters.
